# Evolved changes in reflex control of the cardiovascular system in deer mice native to high altitude

**DOI:** 10.1242/jeb.249483

**Published:** 2025-06-18

**Authors:** Oliver H. Wearing, John J. McGuire, Graham R. Scott

**Affiliations:** ^1^ Department of Biology, McMaster University, 1280 Main Street West, Hamilton, ON, Canada, L8S 4K1; ^2^ Department of Cellular & Physiological Sciences, University of British Columbia, Vancouver, BC, Canada, V6T 2A1; ^3^ Centre for Heart, Lung & Vascular Health, University of British Columbia Okanagan, Kelowna, BC, Canada, V1V 1V7; ^4^ Department of Medical Biophysics, Schulich School of Medicine & Dentistry, Western University, London, ON, Canada, N6A 5C1

**Keywords:** Autonomic control, Systemic vascular resistance, Vasomotor function, High-altitude adaptation, Acclimatization, Mammal

## Abstract

The cold and hypoxic conditions at high altitude place high demands on the cardiovascular system to sustain circulatory O_2_ transport. High-altitude natives have evolved to overcome cold hypoxia, but the cardiovascular mechanisms involved remain poorly understood in most taxa. Here, we investigated the evolved changes in reflex control of cardiovascular function in deer mice (*Peromyscus maniculatus*) native to high altitude. High- and low-altitude populations of deer mice were each bred in captivity and then chronically acclimated to warm normoxia (25°C, ∼20 kPa O_2_) or cold hypoxia (5°C, 12 kPa O_2_) for 6–8 weeks. Cardiovascular function was measured *in vivo* using physiological telemeters, complemented by wire myography to examine vascular function *ex vivo*. High-altitude mice acclimated to cold hypoxia exhibited greater heart rates and were better able to maintain blood pressure in moderate and severe hypoxia, in association with less pronounced depression of metabolism and body temperature. High-altitude mice also exhibited greater baroreflex sensitivity than low-altitude mice across acclimation environments, as reflected by greater changes in heart rate and smaller changes in arterial blood pressure during pharmacological manipulations. Mesenteric arteries from each population exhibited similar *ex vivo* smooth muscle contractions in response to phenylephrine (α_1_-adrenoceptor agonist), and similar endothelium-dependent relaxation in response to acetylcholine, suggesting that evolved changes in the baroreflex arise from adjustments in autonomic control of the heart and/or other resistance vessels. These evolved changes in cardiovascular function and reflex control may be valuable for supporting high metabolic rates in the cold and hypoxic environment at high altitude.

## Teaser

**Summary:** Deer mice native to high altitude have an augmented baroreflex and exhibit stronger cardiovascular responses to hypoxia, which likely helps improve circulatory O_2_ transport in their cold hypoxic environment.

## INTRODUCTION

Cardiovascular function is imperative for the appropriate transport of respiratory gases, nutrients and waste products throughout the body across a broad range of environmental and metabolic conditions. Two vital mechanisms of cardiovascular control in vertebrates are the arterial baroreflex and the hypoxic chemoreflex. The arterial baroreflex exhibits negative feedback control of arterial blood pressure by adjusting cardiac output and systemic vascular resistance. This baroreflex control of arterial blood pressure is vital for maintaining tissue perfusion pressure and O_2_ delivery in a range of conditions. The hypoxic chemoreflex controls the response to reductions in arterial O_2_ tension, including adjustments in tissue blood flow through changes in cardiac output and systemic vascular resistance. These reflexes act and intersect via efferent pathways involving the autonomic nervous system. Although much is known about how these reflexes work, less is known about how they may by modulated by environmental acclimation/acclimatization or evolutionary (genetically based) adaptation to help overcome physiological challenges.

Studies of high-altitude natives present a valuable opportunity to examine this issue. The cold and hypoxic conditions at high altitude require that endotherms maintain high metabolic rates in air with low O_2_ availability, which imposes high demands for circulatory O_2_ transport (McClelland and Scott, 2019; Scott et al., 2024; Storz and Cheviron, 2021). Cardiovascular function is essential for overcoming these challenges, but the plastic and evolved adjustments in cardiovascular function in high-altitude natives remain poorly understood in most taxa. Emerging evidence suggests that the baroreflex can be modified by acclimatization and/or adaptation to high-altitude hypoxia in humans. During acute hypoxia, systemic tissues faced with O_2_ limitation produce local factors that tend to cause vasodilation and reduce peripheral resistance (Kulandavelu et al., 2015; Premont et al., 2020; Weisbrod et al., 2001). Baroreflex stimulation of cardiac output via sympathetic activation is then critical for maintaining arterial blood pressure and meeting the increased demand of tissues for blood flow (Bernardi, 2007). However, chronic activation of the baroreflex can impair baroreflex sensitivity to additional modulators of blood pressure. Indeed, humans native to low altitude exhibit reduced baroreflex sensitivity when exposed to chronic hypoxia at high altitude (Alvarez-Araos et al., 2024; Bourdillon et al., 2017, 2018; Fisher et al., 2022; Steinback et al., 2009) and reduced sympathetic neurovascular transduction (Berthelsen et al., 2020; Simpson et al., 2019). Similarly, in domestic rats, acute hypoxia results in sympathoexcitation and parasympathetic withdrawal and thus impairs baroreflex control (Beltrán et al., 2020). In contrast, Sherpa adapted to high altitudes have greater baroreflex sensitivity in hypoxia than lowlanders, in association with lower baseline sympathetic activity along with greater neurovascular transduction and/or α-adrenergic vasoconstrictive reserve (Bernardi et al., 1998; Berthelsen et al., 2020; Simpson et al., 2019, 2021). Whether similar evolved changes are exhibited in adults of other animal species at high altitude is poorly understood. This is particularly true of small mammals, for which the demands on the circulatory system are expected to be greater than for larger endotherms due to the increased metabolic costs of thermogenesis to cope with the cold temperatures at high altitude.

It also remains unclear whether small high-altitude mammals differ in the overall cardiovascular response to hypoxia, which results from the combined effects of the baroreflex, hypoxic chemoreflex and local factors in peripheral tissues, along with centrally controlled adjustments in metabolism and body temperature. However, important differences in the cardiovascular response to hypoxia between high- and low-altitude taxa have been previously reported in birds. In the low-altitude barnacle goose (*Branta leucopsis*), acute hypoxia reduces peripheral vascular resistance (likely resulting from vasodilation), which is only partially offset by increases in cardiac output such that arterial blood pressure declines (Lague et al., 2016). In contrast, in high-altitude bar-headed geese (*Anser indicus*) and Andean geese (*Chloephaga melanoptera*), reductions in peripheral resistance in acute hypoxia are associated with sufficient increases in cardiac output that arterial pressure is well maintained (Lague et al., 2017). Interestingly, systemic vasodilation in response to acute hypoxia appears to be attenuated over more prolonged exposures to chronic hypoxia in both rats and humans, in concert with sympathetic overactivity and hypertension (Hansen and Sander, 2003; Huang et al., 2002; Lundby et al., 2018; Narvaez-Guerra et al., 2018; Simpson et al., 2024). Whether high-altitude mammals exhibit similar responses to chronic hypoxia as those of their low-altitude counterparts remains poorly understood.

In this study, we address these knowledge gaps by examining reflex control of cardiovascular function in the deer mouse (*Peromyscus maniculatus*), the species with the broadest altitudinal range of any North American mammal. High-altitude populations of this species maintain higher field metabolic rates than their low-altitude counterparts (Hayes, 1989a,b), likely because of the increased costs of thermogenesis in the cold environment at high altitude. Correspondingly, aerobic capacity for thermogenesis is increased in high-altitude deer mice by plastic and evolved adjustments across the O_2_ transport pathway and in thermogenic tissues (Cheviron et al., 2013; Dane et al., 2018; Lui et al., 2015; Rezende et al., 2004; Shirkey and Hammond, 2014; Tate et al., 2017, 2020; Van Sant and Hammond, 2008; Velotta et al., 2016). Key to this increase in thermogenic capacity is corresponding variation in cardiac output and vascular O_2_ transport (Scott et al., 2024; Tate et al., 2020), but potential differences in cardiovascular control remain poorly understood. We previously reported that high-altitude deer mice maintain the capacity for adrenergic vasoconstriction in chronic hypoxia, in contrast to white-footed mice (*P. leucopus*; a congeneric found exclusively at low altitude), in which the vascular responsiveness to α-adrenergic stimulation is blunted (Wearing et al., 2022). These findings provide indirect evidence that there may be evolved changes in baroreflex control in high-altitude mice, but direct tests of baroreflex function were not performed. Furthermore, previous measurements were made in mice held at warm temperatures, at which metabolic demands are low, and did not examine how cardiovascular function and control may be adjusted by chronic exposure to cold combined with hypoxia. In the present study, we tested the hypothesis that reflex control of cardiovascular function is amplified in high-altitude deer mice to better maintain systemic blood pressure and circulatory O_2_ transport in cold hypoxia.

**List of symbols and abbreviations**

EC_50_: effective concentration at 50% of the maximum response
*f*_H_: heart rate
*F*_max_: maximum force produced in response to phenylephrine
*F*_min_: minimum force produced in response to acetylcholine
*G*_50_: baroreflex gain, defined in Eqn 2
*n*: Hill coefficient
*P*_O_2__: partial pressure of oxygen
*P*_mean_: mean arterial pressure
SNP: sodium nitroprusside
*T*_b_: body temperature
*V̇*_O_2__: oxygen consumption rate
Δ: scope of change
Δ*f*_H_: difference between maximum and minimum heart rate
Δ*P*_mean_: difference between maximum and minimum mean arterial pressure

## MATERIALS AND METHODS

### Animals and environmental treatments

Experiments were conducted on deer mice, *Peromyscus maniculatus* (Wagner 1845), raised in captive breeding colonies that we have used in past research (Wearing and Scott, 2022a). The colonies are established from wild mice that are live-trapped at low altitude in the Great Plains (Buffalo County, NE, USA, 40°41′58″N, 99°04′53″W; approximately. 660 m above sea level) or at high altitude near the summit of Mount Blue Sky (formerly ‘Mount Evans’, Clear Creek County, CO, USA, 39°35′18″N, 105°38′38″W; 4350 m above sea level). Wild mice are transported to McMaster University and bred in captivity within their respective population to produce lab-born progeny, which are generally bred and raised in common laboratory conditions (25°C, approximately 20 kPa O_2_). The experiments here used second-generation progeny of 6–10 months of age from nine families of low-altitude mice and 10 families of high-altitude mice (often referred to as ‘lowlanders’ and ‘highlanders’, respectively). Male and female mice from each population were divided at random into two acclimation groups that were exposed to the following environmental conditions for 6–8 weeks: (1) warm normoxia (25°C, ∼20 kPa O_2_), or (2) cold hypoxia (5°C, 12 kPa O_2_) simulating ambient O_2_ levels and winter burrow temperatures at high altitude (Hayward, 1965). Cold hypoxic conditions were maintained using custom-made hypobaric chambers (Lui et al., 2015; McClelland et al., 1998) housed in a temperature-controlled room. All mice were provided with unlimited access to water and standard rodent chow (Teklad 22/5 Rodent Diet formula 8640; Envigo). Cages were cleaned twice weekly, which required that cold hypoxic mice be briefly returned to normobaria (<20 min). All animal procedures followed guidelines established by the Canadian Council on Animal Care and were approved by the McMaster University Animal Research Ethics Board (Animal Use Protocol 20-01-02).

### *In vivo* assessments of the reflex control of cardiovascular function

We measured the reflex control of cardiovascular function in mice for which we have previously reported routine body temperature and cardiovascular function throughout the diel cycle (Wearing and Scott, 2022a). Following 6 weeks in the respective acclimation condition, each animal was surgically instrumented with a physiological telemeter (HD-X11; Data Sciences International, St Paul, MN, USA) to measure arterial blood pressure (in the ascending aortic arch, via the left common carotid artery), heart rate (*f*_H_) and body temperature (*T*_b_). Isoflurane anaesthesia, telemeter placement in the interscapular subcutaneous space, post-surgical pain management (carprofen) and 7-day recovery were conducted as previously described in full detail (Wearing and Scott, 2022a,b). Normal circadian oscillations of mean arterial blood pressure (*P*_mean_), *f*_H_ and *T*_b_ in awake and freely behaving mice became stable after 3–4 days post-surgery. Routine measurements that have been previously reported (Wearing and Scott, 2022a) were then conducted over the next 2–3 days. We then made the two series of *in vivo* measurements reported here: (i) cardiovascular responses to progressive stepwise hypoxia in awake mice; then (ii) pharmacological assessment of baroreflex function in anaesthetized mice.

Responses to progressive stepwise hypoxia were made at 25°C using methods that we have previously described in detail in a study of CD-1 strain domestic house mice (Wearing and Scott, 2022b). Measurements were made in each of the four experimental groups of deer mice: warm normoxic lowlanders, *n*=13 (6 males, 7 females; body mass=25.5±1.6 g mean±s.e.m.); cold hypoxic lowlanders, *n*=12 (5 males, 7 females; 23.6±1.1 g); warm normoxic highlanders, *n*=9 (5 males, 4 females; 21.7±0.6 g); and cold hypoxic highlanders, *n*=12 (6 males, 6 females; 23.3±0.7 g). Briefly, mice were weighed and placed in an open-flow respirometry chamber (530 ml) supplied with 600 ml min^−1^ normoxic air (21% O_2_, balance N_2_). Mice were left for 20–60 min until calm and physiological measurements became stable. The chamber then continued to be supplied with normoxic air for another 20-min measurement period, after which the incurrent O_2_ partial pressure (*P*_O_2__) was reduced stepwise to 16, 12, 10, 9 and 8 kPa O_2_, with a period of 20 min at each *P*_O_2__ step. The first 10 min at each step allowed the mouse to adjust to the new *P*_O_2__, and steady-state resting measurements were then recorded for analysis during the last 10 min of each step. Occasional brief periods of heightened activity during this recording window were excluded from the selections used for analysis. Incurrent chamber flow rate, incurrent and excurrent O_2_ and CO_2_ fractions, arterial blood pressure and subcutaneous temperature (a proxy for *T*_b_) were acquired at 1 kHz using a PowerLab 16/32 data acquisition unit and Labchart 8 Pro software (ADInstruments, Colorado Springs, CO, USA) with PhysioTel Connect (ADInstruments) for acquisition of telemetry data via an MX2 data acquisition system (Data Sciences International). Oxygen consumption rate (*V̇*_O_2__) was calculated at standard temperature and pressure (STP) using established equations for incurrent flow measurement (Lighton, 2018), and is expressed relative to body mass. *P*_mean_ and *f*_H_ were calculated from the systolic–diastolic oscillations in arterial pressure.

Measurements of baroreflex function were then conducted the day after measuring cardiovascular responses to progressive stepwise hypoxia. Mice were anaesthetized to a surgical plane using 2–3% isoflurane (delivered in 100% O_2_), and the right external jugular vein was catheterized with a 5-cm length of saline-filled heparinized microrenathane tubing (MRE025, Braintree Scientific, Braintree, MA, USA) for direct intravenous administration of pharmacological agents (as described below). Once surgery was completed, blood pressure and heart rate data were recorded via the telemeter, and the isoflurane dose was held at the minimum level at which there was a <10-mmHg *P*_mean_ response to toe pinch (1.5–2.0% isoflurane in O_2_). This low dose of isoflurane is widely considered the best choice of anaesthesia for studies measuring cardiovascular function and regulation in rodents (Janssen et al., 2004; Lorenz, 2002; Wearing et al., 2025), minimizing the potential influence of anaesthetics on cardiovascular function and control, while avoiding the confounding effects of variation in animal activity and metabolism. At this isoflurane level, *P*_mean_ and *f*_H_ remained stable in most individuals at levels resembling resting conscious values, suggesting that autonomic control of arterial pressure remained functionally intact across experimental groups. However, values did not stabilize in a small subset of animals that were thus excluded from the final dataset, such that the final sample sizes for each group were: warm normoxic lowlanders, *n*=11 (5 males, 6 females); cold hypoxic lowlanders, *n*=10 (4 males, 6 females); warm normoxic highlanders, *n*=8 (4 males, 4 females); and cold hypoxic highlanders, *n*=8 (3 males, 5 females).

Baroreflex function was assessed by measuring the changes in *f*_H_ and *P*_mean_ after intravenous injections of phenylephrine (an α-adrenergic vasopressor that increases *P*_mean_ and causes a baroreflexive decrease in *f*_H_) followed by sodium nitroprusside (SNP; a vasodilatory nitric oxide donor that decreases *P*_mean_ and causes a baroreflexive increase in *f*_H_). Baseline *P*_mean_ and *f*_H_ data were collected for 5 min to ensure stable measures. Serial doses of phenylephrine (phenylephrine hydrochloride; Sigma-Aldrich, Oakville, ON, Canada) of ascending concentrations (0.15, 1.5, 15 and 150 µg ml^−1^) were then injected intravenously in 3.33-µl injections per gram of animal mass, such that mice received 0.5, 5, 50 and 500 µg kg^−1^ doses of phenylephrine in series, with 1- to 2-min washout periods between doses before *P*_mean_ and *f*_H_ returned to pre-injection values. Similar increasing concentrations (0.3, 3, 30 and 300 µg ml^−1^) of SNP (Sigma-Aldrich) were subsequently injected at 3.33 µl g^−1^ to final doses of 1, 10, 100 and finally 1000 µg kg^−1^ (with 3- to 5-min washout periods between each dose). The highest doses of each pharmacological agent were generally found to be saturating, as reflected by a lack of response compared to the second highest dose. Mice were then euthanized by cervical dislocation, weighed and dissected for organ mass measurements that have been reported previously (Wearing and Scott, 2022a).

Baroreflex curves were generated using the *P*_mean_ and *f*_H_ values recorded following phenylephrine and SNP injections. We took the highest *P*_mean_ and lowest *f*_H_ values following each phenylephrine injection (which occurred within 30 s) and the lowest *P*_mean_ and highest *f*_H_ values following each SNP injection (which occurred within 1 min), ensuring the response to mounting doses increased and then plateaued without incidence of tachyphylaxis. We then fitted the following asymmetrical baroreflex curve (Filogonio and Leite, 2023) to the data for each individual mouse:
(1)

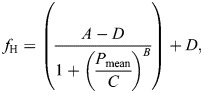
where *A* is overall maximum *f*_H_, *D* is overall minimum *f*_H_, *C* is the midpoint value of the *P*_mean_ response range and *B* is the operating range coefficient. Curves were fitted to the data using GraphPad Prism 10 (GraphPad Software LLC, Boston, MA, USA). We used the coefficients from the asymmetrical baroreflex curve to calculate baroreflex gain, *G*_50_, for each individual using Eqn 2 (Wong et al., 1993):
(2)

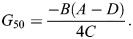


Here, we report *G*_50_, the overall maximum and minimum *f*_H_ and *P*_mean_ values obtained at the highest doses of phenylephrine and SNP, and the difference between these overall maximum and minimum values (Δ*f*_H_ and Δ*P*_mean_) for each individual mouse.

We also determined an average baroreflex curve for each experimental group to visually compare mean baroreflex function between groups. This was accomplished using the baroreflex curve for each individual to calculate its curve-predicted *f*_H_ values across the full *P*_mean_ range from 0 to 200 mmHg, and then fitting an asymmetrical baroreflex curve to the entire set of resulting data from all the mice in the group. These average baroreflex curves as well as the baroreflex curves for each individual mouse are shown in Fig. S1.

### *Ex vivo* assessments of vascular reactivity using wire myography

We assessed vascular reactivity by wire myography in isolated resistance arteries from the mesenteric vascular bed using treatments of phenylephrine (α_1_-adrenoceptor agonist and vasoconstrictor) and acetylcholine (muscarinic receptor agonist and endothelium-dependent vasodilator). We chose to focus our assessments on a separate cohort of (non-telemetered) warm normoxic mice (*n*=7 lowlanders, 3 males, 4 females; *n*=8 highlanders, 4 males, 4 females), based on observations from the experiments described above that *in vivo* baroreflex function differed between populations but was unaffected by acclimation environment. We used a two-channel wire myography system (DMT Model 620M, Danish Myo Technology A/S, Hinnerup, Denmark) and a PowerLab 2/25 data acquisition unit running Labchart 8 Pro software (ADInstruments) to measure and record isometric tension of arterial ring preparations as we briefly describe here. Deer mice were euthanized by isoflurane overdose followed by cervical dislocation, and the intestinal tract containing the mesenteric vasculature was removed from the abdomen. First-order branches of the superior mesenteric artery were isolated and cut into two replicate arterial ring segments [mean±s.d.: length, 1.49±0.32 mm; diameter (normalized at a 7.98 kPa target pressure), 114±25 µm] that were separately mounted in the myograph chambers using 20-µm (diameter) gold-plated tungsten wire. Within these chambers, vessels were bathed in Krebs-HEPES buffer adjusted to pH 7.4 (Wang et al., 2022) that was continuously bubbled with carbogen (95% O_2_, 5% CO_2_), and temperature was maintained at 37°C. Resting tension for each vessel was set to 90% of the diameter estimated to produce wall stress equivalent to 7.98 kPa (Kagota et al., 2011; McGuire et al., 2007), and vessels were allowed to equilibrate for 20 min in the chambers in fresh buffer, with baseline tension recorded. Vessels were then bathed with 80 mmol l^−1^ KCl dissolved in Krebs-HEPES buffer to confirm viability and determine the force produced by a standardized contractile stimulus for each vessel, and to provide a value against which subsequent contractions to phenylephrine (see below) could be normalized. Vessels were then rinsed and allowed to relax for 20 min in fresh buffer so that vessel tension returned to baseline. These washout periods were repeated between the treatment protocols for blood vessel contractility and endothelium-dependent relaxations. The final concentrations of compounds in the baths are reported here. Blood vessel contractility was assessed by measuring the increase in tension produced after addition of increasing cumulative concentrations of phenylephrine (1 nmol l^−1^ to 50 µmol l^−1^) at which point tension began to plateau, i.e. *F*_max_. Endothelium-dependent relaxations were assessed by pre-contracting the vessels with 50 µmol l^−1^ phenylephrine, then introducing ascending cumulative doses of acetylcholine (1 nmol l^−1^ to 10 µmol l^−1^). Vessels were then bathed in 1 mmol l^−1^ SNP to elicit maximal relaxations and provide a range for normalization of relaxations to acetylcholine. Contractions to phenylephrine, as reflected by the increase in tension from baseline, were expressed relative to the force produced above baseline by contraction with 80 mmol l^−1^ KCl (% contraction). Relaxation responses to acetylcholine, as reflected by the decrease in tension relative to pre-constriction with phenylephrine, were expressed relative to the difference in tension between pre-constriction with 50 µmol l^−1^ phenylephrine and following incubation with 1 mmol l^−1^ SNP (as % relaxation). For each animal, data from each of the two replicate vessels were averaged, and dose–response curves were then fitted to the data for each animal using Eqn 3:
(3)

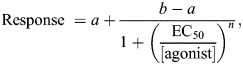
where ‘Response’ is the % contraction or % relaxation caused by the concentration of phenylephrine or acetylcholine (i.e. [agonist], on the *x*-axis), respectively; *a* and *b* refer to the lower and upper plateaus in the Response (i.e. *y*-) axis, respectively; EC_50_ is the effective (molar) concentration at 50% of *F*_max_ or *F*_min_, and *n* is the Hill coefficient (i.e. steepness of the curve). The ‘Response’ data along with the maximum/minimum force (*F*_max_ for phenylephrine, *F*_min_ for acetylcholine; i.e. the *b* value in Eqn 3) and EC_50_ values for each individual mouse are reported in Fig. 3 and Table 3. The mean coefficient values for each population were calculated as the average of the coefficients generated for each individual using Eqn 3. Data points from the resulting curve equations from each individual were then used to generate an average fitted curve for each population (similarly to average baroreflex curves described above), and these were then compared between populations to see whether responses for both populations could be characterized by a single curve (see Statistical analysis). If so, a single curve was generated from the data from both populations combined. All curves were fitted using GraphPad Prism.

### Statistical analysis

Statistical comparisons were made using linear mixed models with the lme4 package (Bates et al., 2015) in R v.4.4.1 (https://www.r-project.org/). Data from males and females were included in our analyses, with both sex and family included as random factors in all linear models. For baroreflex data, we tested for the main effects of population (lowland versus highland) and acclimation environment (warm normoxia versus cold hypoxia) as well as the interactions between these factors. Holm-adjusted Tukey's HSD *post hoc* tests were performed to test for the pairwise differences between acclimation environments within each population, or between populations within each acclimation environment. For the responses to progressive stepwise hypoxia, we tested for effects of and interactions between population, acclimation environment and inspired *P*_O_2__. Body mass was included as a covariate when *P*<0.10 (or excluded in final models if *P*>0.10 in initial tests). For each population, Holm-adjusted Tukey's HSD *post hoc* tests were performed to test for the pairwise differences between acclimation environments within each *P*_O_2__, and between each *P*_O_2__ versus the initial value at 21 kPa within each acclimation group. For wire myography experiments, data from males and females were combined, and two-tailed unpaired *t*-tests with Welch's correction were performed to test for effects of population on equation constants. Absolute values were used for these analyses, with the exception of EC_50_ data, which were log_10_ transformed before analysis (though we report absolute values). Extra sum-of-squares *F*-tests were also performed to compare dose–response curves between populations using an α value of 0.05. Statistical analyses were generally carried out on absolute values of traits that were not corrected for body mass (because effects of body mass were accounted for as a covariate in statistical models when *P*<0.10), but the *V̇*_O_2__ data presented here are expressed relative to body mass as is conventional in the literature. Data are presented as individual values, sometimes along with box and whisker plots, or as means±s.e.m.

## RESULTS

### Acclimation to cold hypoxia blunted hypotension in acute hypoxia, particularly in highlanders

**Fig. 1.**
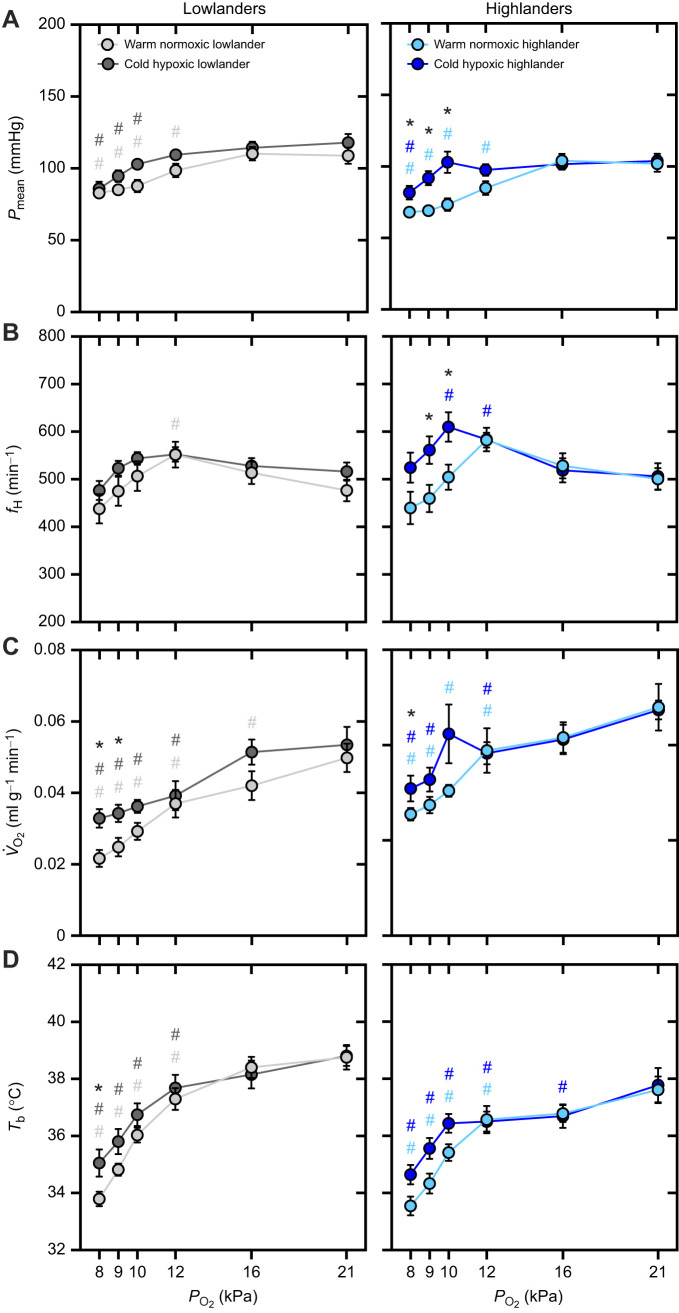
**Highland deer mice exhibited better maintenance of blood pressure and a more pronounced heart rate response to severe hypoxia after acclimation to cold hypoxia.** The responses of mean arterial pressure (*P*_mean_; A), heart rate (*f*_H_; B), oxygen consumption rate (*V̇*_O_2__; C) and body temperature (*T*_b_; D) to acute stepwise hypoxia were measured in lowland (grey, left panels) and highland (blue, right panels) deer mice that were chronically exposed to warm normoxia (lighter shades) or cold hypoxia (darker shades). Data are shown as means±s.e.m. with sample sizes as follows: warm normoxic lowlanders, *n*=13 mice; cold hypoxic lowlanders, *n*=12 mice; warm normoxic highlanders, *n*=9 mice; and cold hypoxic highlanders, *n*=12 mice. Statistical results from linear mixed models are shown in Table 1. Pairwise differences were assessed using Holm-adjusted Tukey's HSD tests for multiple comparisons: # represents a significant (*P*<0.05) pairwise difference compared with the value at 21 kPa within that acclimation group (lighter shaded symbols for warm normoxia groups, darker shaded symbols for cold hypoxia groups), and * represents a significant (*P*<0.05) pairwise difference between acclimation groups at that *P*_O_2__.

We measured the responses to progressive stepwise hypoxia to consider blood pressure maintenance and heart rate responses under the combined influence of the baroreflex, hypoxic chemoreflex, local factors in peripheral tissues and changes in metabolism (Fig. 1). In general, exposure to acute hypoxia had a strong effect on all measured traits (main effects of *P*_O_2__, *P*<0.001; Table 1). Mean arterial pressure (*P*_mean_; Fig. 1A) was maintained and heart rate (*f*_H_; Fig. 1B) tended to increase in mild to moderate hypoxia, but *P*_mean_ declined in severe hypoxia as *f*_H_ returned towards baseline values. The declines in *P*_mean_ in severe hypoxia were often associated with reductions in oxygen consumption rate (*V̇*_O_2__; Fig. 1C) and body temperature (*T*_b_; Fig. 1D).

**Table 1.** Statistical results of linear mixed models of the cardiovascular responses to progressive hypoxia

Trait	Body mass effect	Population (P) effect	Acclimation (A) effect	*P*_O_2__ effect	P×A	P×*P*_O_2__	A×*P*_O_2__	P×A×*P*_O_2__
*P*_mean_	*P=0.068*	*P=0.059*	***P*=0.003**	***P*<0.001**	*P*=0.132	*P*=0.910	***P*<0.001**	*P=0.060*
*f*_H_	n.s.	*P*=0.702	*P*=0.165	***P*<0.001**	*P*=0.262	*P*=0.666	***P*<0.001**	*P=0.098*
*V̇*_O_2__	***P*=0.007**	*P*=0.726	***P*=0.013**	***P*<0.001**	*P*=0.845	*P=0.070*	*P*=0.137	*P*=0.509
*T*_b_	n.s.	***P*=0.010**	*P=0.070*	***P*<0.001**	*P*=0.954	***P*=0.022**	***P*=0.002**	*P*=0.945

*P*_mean_, mean arterial blood pressure; *f*_H_, heart rate; *V̇*_O_2__, oxygen consumption rate; *T*_b_, body temperature. n.s. denotes a non-significant effect of body mass (*P*≥0.10), which was subsequently removed as a covariate from the linear model to calculate the other *P*-values shown. Significant effects (*P*<0.05) are shown in bold, and close to significant effects (*P*<0.10) are italicized.

Acclimation to cold hypoxia attenuated hypotension in severe hypoxia, in contrast to the pronounced hypotension observed in warm normoxic mice, but this effect seemed to be driven primarily by variation in highlanders. There was a significant effect of acclimation on *P*_mean_ (acclimation effect, *P*=0.003) along with a significant acclimation×*P*_O_2__ interaction (*P*<0.001). This was associated with cold hypoxic highlanders exhibiting significantly greater *P*_mean_ than warm normoxic highlanders when *P*_O_2__ was ≤10 kPa, such that *P*_mean_ was maintained at normoxic levels (i.e. values at 21 kPa O_2_) in all but the most severe level of hypoxia (Fig. 1A). In contrast, there were no significant effects of cold hypoxia acclimation on *P*_mean_ in lowlanders. The improved maintenance of *P*_mean_ in highlanders after acclimation was associated with a pronounced increase in *f*_H_ in severe hypoxia (Fig. 1B). This response likely drove the significant acclimation×*P*_O_2__ interaction on this trait (*P*<0.001), because there was no effect of acclimation on *f*_H_ in lowlanders. Acclimation to cold hypoxia also appeared to reduce the depression of *V̇*_O_2__ and *T*_b_ in severe hypoxia (Fig. 1C,D). Differences in the effects of acclimation between highlanders and lowlanders were less apparent for these traits, although only cold hypoxic highlanders maintained *V̇*_O_2__ at normoxic levels at 10 kPa O_2_. There was also a significant population effect on *T*_b_ (*P*=0.010), which was lower in highlanders than in lowlanders in normoxia and moderate hypoxia.

### Highland deer mice have a greater baroreflex sensitivity than lowland deer mice

We measured the *f*_H_ and *P*_mean_ responses to phenylephrine followed by SNP in anaesthetized mice to uncover potential evolved and/or environmentally plastic changes in baroreflex function. We measured the maximum and minimum *P*_mean_ and *f*_­H_ values recorded throughout the baroreflex assessments, and used these to calculate the scope of change (Δ) in each of these indices of cardiovascular function (Δ*P*_mean_ and Δ*f*_H_, respectively). Population differences were greatest after saturating phenylephrine administration, when highlanders exhibited lower values of maximum *P*_mean_ (population effect, *P*=0.003; Table 2) and minimum *f*_H_ (*P*=0.041). Minimum *P*_mean_ and maximum *f*_H_ elicited by saturating SNP were similar between populations (*P*=0.738 and *P*=0.940, respectively). As such, the difference between maximum and minimum values of *P*_mean_ (Δ*P*_mean_) was 17–21% lower on average in highlanders versus lowlanders (population effect, *P*=0.001). The population effect on the difference between maximum and minimum values of *f*_H_ (Δ*f*_H_) neared significance (*P*=0.071) owing to 2.1-fold higher average values in highlanders versus lowlanders in warm normoxia.

**Table 2.** Cardiovascular parameters following subcutaneous injections of saturating phenylephrine (post-PE) and saturating sodium nitroprusside (post-SNP) during assessments of baroreflex function in anaesthetized deer mice

	Lowlanders		Highlanders		
	Warm normoxia	Cold hypoxia	Warm normoxia	Cold hypoxia	*P*<0.05^a^
*n*	11	10	8	8	
*P*_mean_ (mmHg)					
Maximum, post-PE	140±3	136±4	123±2*	126±6	P
Minimum, post-SNP	62±3	61±2	61±2	64±3	–
Δ*P*_mean_	78±3	75±5	62±2*	62±5	P
*f*_H_ (min^−1^)					
Maximum, post-SNP	587±13	618±13	573±18	620±25	A
Minimum, post-PE	474±21	460±28	332±27*	438±44	P; P×A
Δ*f*_H_	113±16	159±19	240±30*	182±56	P; P×A

*Significant pairwise difference between warm normoxic highlanders and warm normoxic lowlanders. ^a^Letters below reflect significant main effects of population (P), acclimation (A) and/or their interaction (P×A). Data are presented as means±s.e.m.

**Fig. 2.**
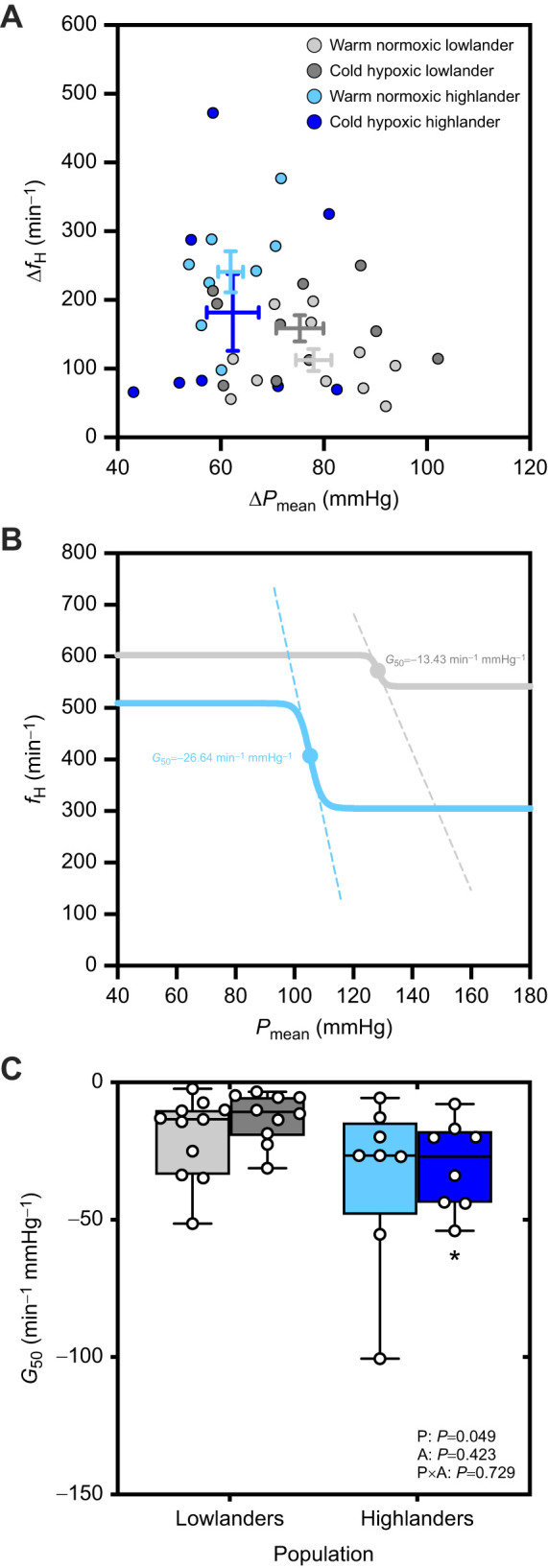
**Baroreflex sensitivity was greater in highland deer mice (blue) than in lowland deer mice (grey), in measurements of mice acclimated to warm normoxia (lighter shades) or cold hypoxia (darker shades).** (A) The difference between maximum and minimum values of *f*_H_ (Δ*f*_H_) and *P*_mean_ (Δ*P*_mean_) during pharmacological manipulation with saturating doses of phenylephrine and sodium nitroprusside, shown as means±s.e.m. and circles for each individual. (B) Representative curves (bold solid curves) of a warm normoxic lowlander and a warm normoxic highlander, with the dashed lines showing the baroreflex gain (*G*_50_, or the slope at the midpoint value of the *P*_mean_ response range, shown by the central circle on each curve) for each individual. (C) Baroreflex gain was calculated from the baroreflex curve for each individual (Fig. S1), and is shown using box and whisker plots with individual data shown as circles. Data from each individual are shown, with sample sizes of each group as follows: warm normoxic lowlanders, *n*=11 mice; cold hypoxic lowlanders, *n*=10 mice; warm normoxic highlanders, *n*=8 mice; and cold hypoxic highlanders, *n*=8 mice. Statistical results from linear mixed models (two-tailed) are shown for the effects of population (P), acclimation (A) and their interaction (P×A), where *P*<0.05. *Significant pairwise difference between populations within an acclimation environment (*P*<0.05), as assessed by Holm-adjusted Tukey's HSD tests (two-tailed) for multiple comparisons.

Consideration of the combined changes in *P*_mean_ and *f*_H_ suggested that baroreflex sensitivity was greater in highland mice (Fig. 2). There was an upward and leftward shift in the relationship between Δ*f*_H_ and Δ*P*_mean_ in highlanders compared with lowlanders, reflecting an exaggerated *f*_H_ response and reduced change in pressure in response to a baroreflex disturbance (Fig. 2A). To visualize the full baroreflex-dependent relationship between blood pressure and heart rate, we generated baroreflex curves using absolute *P*_mean_ and *f*_H_ data recorded throughout the pharmacological baroreflex assessment for each individual (Fig. S1). Representative curves for a high-altitude mouse and a low-altitude mouse are shown in Fig. 2B. Consistent with the aforementioned population differences in Δ*f*_H_ and Δ*P*_mean_, these baroreflex curves show a greater and steeper *f*_H_ response to changes in *P*_mean_ in the highlander compared with the lowlander (Fig. 2B). From these curves, baroreflex gain (*G*_50_) was calculated as the slope of the curve at the midpoint of the *P*_mean_ response range (i.e. dotted lines in Fig. 2B). This index of baroreflex sensitivity was greater in magnitude (i.e. more negative) in highlanders than in lowlanders (population main effect, *P*=0.049; Fig. 2C), again indicating that highland mice exhibit greater baroreflex sensitivity than lowland mice.

### Vasomotor sensitivity of mesenteric arteries was similar between populations

**Fig. 3.**
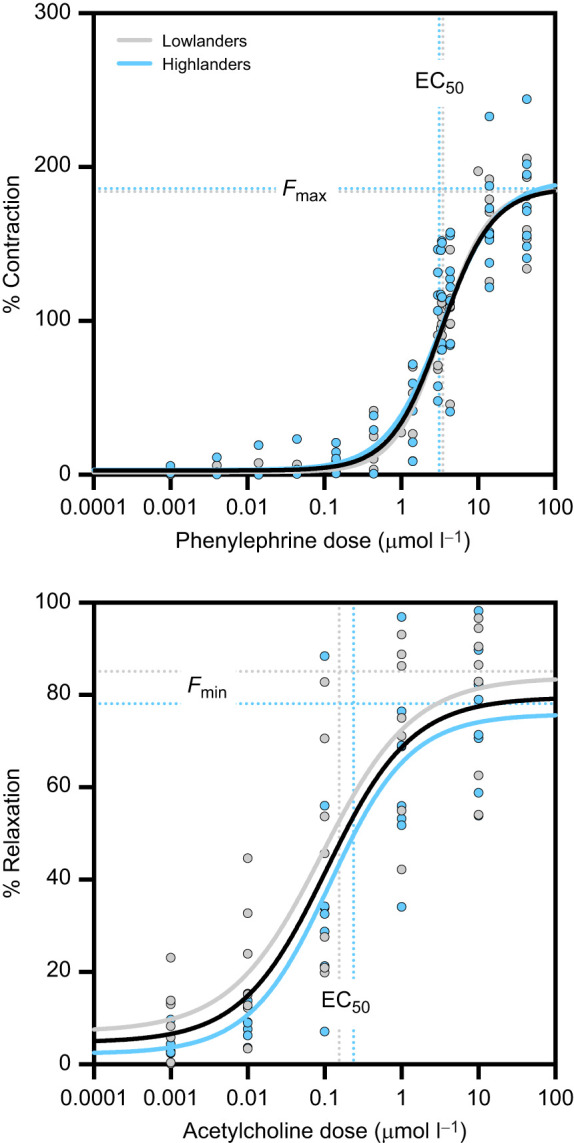
**Vascular responses of isolated mesenteric arteries to phenylephrine and acetylcholine were similar between lowlanders (grey, *n*=7 mice) and highlanders (blue, *n*=8 mice) acclimated to warm normoxia.** (A) α_1_-Adrenoceptor mediated contractions in response to increasing doses of phenylephrine. (B) Endothelium-dependent relaxations in response to increasing doses of acetylcholine. Circles show data points for each individual along with group-average curves produced for each population, with the solid black curves showing the average curve derived from all mice from both populations (see Materials and Methods for curve fitting details). The average maximal responses (*F*_max_ for phenylephrine and *F*_min_ for acetylcholine) and the average effective concentrations at 50% of the maximal response (EC_50_) for each pharmacological treatment for each population are shown as dotted lines. There was no significant difference between the dose–response curves of highlanders versus lowlanders based on results of sum-of-squares *F*-tests (see Materials and Methods).

We examined vasomotor sensitivity to α_1_-adrenoceptor stimulation as a potential mechanism for population differences in baroreflex function. We focused on population comparisons in warm normoxic mice because population effects were the dominant source of variation in baroreflex function (Fig. 2, Table 2). Isolated mesenteric arteries contracted in response to phenylephrine in a dose-dependent manner (Fig. 3A). We also examined endothelium-dependent relaxations in response to acetylcholine (Fig. 3B). We found that the responses to each of these drugs were very similar between highlanders and lowlanders, with a single curve for each drug being sufficient to describe the dose response of both populations (see black curves in Fig. 3). As a result, we also saw no population differences in either the maximal response (*F*_max_ or *F*_min_) or the effective concentration at 50% of the maximal response (EC_50_) for either pharmacological treatment (Table 3). As such, sum-of-squares *F*-tests showed that there was no population difference in either the contraction or relaxation response curves.

**Table 3.** Coefficients from dose–response curves of mesenteric artery reactivity to phenylephrine (contraction) or acetylcholine (relaxation) assessed using wire myography

Coefficient	Lowlanders (*n*=7)	Highlanders (*n*=8)	*P*	All mice
Phenylephrine				
EC_50_ (µmol l^−1^)	3.48±0.70	3.11±0.67	0.597	3.35
*F*_max_ (% contraction)	184±14	186±12	0.929	186
Hill coefficient, *n*	1.61±0.12	1.52±0.23	0.732	1.31
Acetylcholine				
EC_50_ (µmol l^−1^)	0.15±0.05	0.24±0.08	0.448	0.105
*F*_min_ (% relaxation)	85±5	78±5	0.359	80
Hill coefficient, *n*	0.87±0.13	1.18±0.22	0.251	0.79

*F*_max_ and *F*_min_ represent the maximal responses to phenylephrine and acetylcholine, respectively, equal to *b* in Eqn 3 shown in the Materials and Methods. ‘All mice’ represents the average coefficient across both populations, corresponding to the black dose–response curves in Fig. 3. *P*-values were acquired by comparing populations using two-tailed unpaired *t*-tests with Welch's correction (EC_50_ values were log_10_ transformed). Data are presented as means±s.e.m.

## DISCUSSION

Our findings show that highland deer mice have altered reflex control of cardiovascular physiology compared with low-altitude conspecifics. Highland deer mice mounted an augmented heart rate response to progressive hypoxia to better maintain blood pressure after acclimation to cold hypoxia, and had a more responsive and effective baroreflex. Vascular sensitivities to α_1_-adrenoceptor activation and capacity for endothelium-dependent relaxation of small calibre mesenteric arteries did not differ between highland and lowland mice, reflecting preserved responsiveness to regulators of vascular tone in these vessels. These findings suggest that evolved changes in reflex control may arise from adjustments in autonomic control of the heart and/or other resistance vessels.

### Cardiovascular responses to progressive hypoxia in high-altitude deer mice

The cardiovascular responses to hypoxia appear to have evolved in highland deer mice to help support circulatory O_2_ delivery and better maintain O_2_ demands during chronic hypoxia exposure (Fig. 1). With tissue hypoxia, paracrine factors released by peripheral tissues favour vasodilation to increase local blood flow and help restore the balance between O_2_ supply and demand (Kulandavelu et al., 2015; Premont et al., 2020; Weisbrod et al., 2001). However, with global hypoxaemia, systemic vasodilation may reduce *P*_mean_ to such an extent that tissue perfusion pressure and therefore O_2_ delivery (and aerobic metabolism) is constrained. The overall cardiovascular response to hypoxia results from the combined influence of these peripheral factors along with hypoxic chemoreflex activation, the baroreflex, and centrally controlled adjustments in metabolism and body temperature. Here, we observed that mild hypoxia at 16 kPa O_2_ tended to increase *f*_H_, likely reflecting the stimulatory effects of local vasodilatory factors combined with the hypoxic chemoreflex and baroreflex, and *P*_mean_ was maintained at normoxic values (Fig. 1). Declines in *P*_mean_ became apparent in moderate to severe hypoxia (8–12 kPa O_2_), particularly in mice acclimated to warm normoxia, suggesting that the reflex stimulation of cardiac output (including *f*_H_) was no longer able to fully offset the effects of tissue vasodilatory factors. However, cold hypoxic highlanders maintained greater *f*_H_ at 9–10 kPa O_2_, in association better maintenance of blood pressure and aerobic metabolism (i.e. *V̇*_O_2__). These levels of hypoxia are representative of what could realistically be experienced within underground burrows. The ability to maintain blood pressure to support circulatory O_2_ transport at these levels of hypoxia could therefore confer marked advantages in the wild, given the vital importance of aerobic metabolism for thermogenesis and fitness at high altitude (Hayes and O'Connor, 1999; Scott et al., 2024; Wearing and Scott, 2021). Multiple circulatory and metabolic adjustments likely contribute to the variation in cardiovascular function observed here (Chappell et al., 2007; Tate et al., 2020). For example, we have previously found that cold hypoxia acclimation increases maximal stroke volume in highlanders but not in lowlanders (Tate et al., 2020). This increase may enable highlanders to better maintain cardiac output and circulatory O_2_ transport in their cold hypoxic environment, helping avoid hypoxia-induced reductions in *P*_mean_ that could limit tissue perfusion and aerobic metabolism.

The most severe levels of hypoxia led to larger declines in *f*_H_ and *P*_mean_ across groups, and were associated with pronounced declines in aerobic metabolism and body temperature. Reductions in metabolism and body temperature are a common response to acute hypoxia in small mammals, and likely help avoid a mismatch between O_2_ supply and demand during hypoxia-induced hypoxaemia (Frappell et al., 1992; Gu and Jun, 2018). These reductions may have had a significant influence on *f*_H_ here, because hypometabolism likely reduced circulatory O_2_ demands whereas *T*_b_ depression may have reduced intrinsic heart rate by slowing cardiac pacemaker currents (Joyce and Wang, 2022; Senges et al., 1979). Furthermore, studies in ectotherms show that baroreflex sensitivity is strongly affected by body temperature (Sandblom et al., 2016; Zena et al., 2015), so the reductions in *T*_b_ observed here could contribute to why barostatic regulation failed to maintain blood pressure in severe hypoxia. However, metabolic depression did not occur in cold hypoxic highlanders at 10 kPa O_2_, in contrast to all other groups, and highlanders also appeared to exhibit less *T*_b_ depression at this *P*_O_2__ (Fig. 1). This is consistent with previous findings that hypoxia acclimation leads to greater attenuation of *T*_b_ depression in severe hypoxia in highlanders than in lowlanders (Ivy and Scott, 2017), and that this difference may be partly underlain by a non-synonymous mutation in *Epas1*, the gene that encodes hypoxia-inducible factor 2α (Ivy et al., 2022).

### Evolved changes in baroreflex function in high-altitude deer mice

The greater ability of highlanders to maintain *P*_mean_ and *f*_H_ in severe hypoxia could be partly attributed to their greater baroreflex sensitivity (i.e. gain), characterized by greater swings in heart rate in association with smaller changes in arterial pressure compared with low-altitude conspecifics (Fig. 2, Table 2). These measurements necessitated the use of anaesthesia, which can affect baroreflex function, but the low level of isoflurane used here is commonly accepted as the best choice for assessing cardiovascular physiology in rodents (Janssen et al., 2004; Lorenz, 2002; Wearing et al., 2025). At this level of anaesthesia (in absence of phenylephrine/SNP), blood pressure and heart rate are maintained at resting conscious values, and the confounding effects of variation in activity and metabolism are avoided. Furthermore, the modest levels of anaesthesia were consistent across groups, and could not explain the observed differences in baroreflex function between populations. These population differences in baroreflex sensitivity could be partly explained by variation in β_1_-adrenergic responsiveness, as suggested by previous findings that highland deer mice have a greater capacity to increase heart rate in response to β_1_-adrenergic stimulation than lowland white-footed mice (Wearing et al., 2022). This observation could result from increased expression and/or sensitivity of cardiac β-adrenoceptors in highland deer mice. If so, this difference would contrast findings in high-altitude plateau pika, which have lower cardiac β_1_-adrenoceptor expression than domestic rats (a species that is generally found only at low altitudes) (Pichon et al., 2013), but it is unclear whether these findings reflect a specific adaptation to high altitude or a broad taxonomic difference between rodents and lagomorphs such as pika. Chronic hypoxia typically results in reduced expression and desensitization of cardiac β_1_-adrenoceptors in low-altitude taxa, likely in response to prolonged sympathetic activation via the hypoxic chemoreflex, with reductions in the chronotropic effects of pharmacological receptor stimulation that would impair baroreflex potency (Antezana et al., 1992; Browne et al., 1997; Richalet, 2016; Richalet et al., 2024, 1988; Voelkel et al., 1981). Whether similar responses occur during combined exposure to cold and hypoxia is unknown, but such responses did not elicit any significant acclimation-induced changes in the baroreflex here (Fig. 2).

Population differences in the baroreflex could also result from variation in vagal (parasympathetic) control. Our previous work in conscious deer mice under routine conditions showed no population difference in routine vagal chronotropic tone, but chronic exposure to cold hypoxia reduced vagal tone in both populations, leading to increased heart rates to support the metabolic demands of thermogenesis at low temperatures (Wearing and Scott, 2022a). Nevertheless, highlanders could experience greater vagal inhibition during baroreceptor loading compared with lowlanders, which could help explain the trend for highlanders to exhibit greater changes in heart rate. Future assessments of intrinsic heart rate and different components of the baroreflex (e.g. baroreceptor sensitivity, sympathetic and parasympathetic activity, etc.) would provide further clarity on the mechanisms underlying evolved changes in baroreflex control.

Population differences in baroreflex control did not appear to be associated with any variation in vascular sensitivity to α-adrenergic stimulation. This is supported by our finding that mesenteric arteries from highlanders exhibit similar contraction responses to phenylephrine as those from lowlanders in comparisons among warm normoxic mice (Fig. 3). However, it is possible that our findings in first-order mesenteric branches are not fully representative of all other resistance vessels, as vasomotor sensitivity can vary with vessel size and branch order (Bund et al., 1994; Hilgers and De Mey, 2009). Whether higher-order mesenteric resistance vessels or resistance vessels supplying other peripheral capillary beds (e.g. in skeletal muscle) have differences in vasomotor sensitivity in highlanders remains to be seen.

We also found that the capacity for endothelium-dependent relaxation of small mesenteric arteries did not differ between highland and lowland deer mice (Fig. 3, Table 3). Further exploration of the mechanisms underlying endothelium-dependent relaxation is warranted because different local mediators of these responses (e.g. nitric oxide and endothelium-dependent hyperpolarization) have been shown to be altered by hypoxia and may compensate when other vasodilator mechanisms are impaired (Spilk et al., 2013). Heterogeneity in the contribution of different endothelium-dependent mechanisms to muscarinic receptor agonist-induced relaxations of vascular smooth muscle has been described in mouse mesenteric arteries (Zhang et al., 2025). Accordingly, our experiments with first-order mesenteric arteries assessed contributions to endothelium-dependent relaxations by pathways including and not including nitric oxide (e.g. endothelium-dependent hyperpolarization). It is possible that the relative importance of these distinct mechanisms varies in smaller diameter mesenteric vessels. Furthermore, many studies using stimuli other than acetylcholine (e.g. shear stress, other G-protein coupled receptor agonists) have reported variation in endothelium-dependent vasodilation mechanisms. Therefore, a wider selection of endothelial cell agonists and comparative studies across microvasculature beds would enable broader generalization about the mechanisms of endothelium function. There could also be intrinsic population differences in vascular network structure that affected responses to vasomotor substances *in vivo*, based on findings that highlanders have greater capillary densities than lowlanders in some tissues (skeletal muscle) (Lui et al., 2015). Whether such differences affect systemic vascular resistance is unclear, particularly when considering that routine *f*_H_ and *P*_mean_ are similar between populations (Wearing and Scott, 2022a).

### Cardiovascular mechanisms of high-altitude adaptation

High-altitude deer mice exhibit several physiological adjustments that help circumvent the challenges presented by the cold and hypoxic conditions in their native environment, adjustments that arise via the interactive effects of evolutionary adaptation and environmentally induced plasticity (McClelland and Scott, 2019; Scott et al., 2024; Storz and Cheviron, 2021). Highlanders thus have a greater aerobic capacity for thermogenesis than lowlanders, underlain by greater maximal cardiac output along with several other evolved changes across the O_2_ transport pathway and in the metabolic function of thermogenic tissues (Chappell et al., 1988; Coulson et al., 2021; Dane et al., 2018; Hayes and O'Connor, 1999; Lui et al., 2015; Mahalingam et al., 2017; Rezende et al., 2004; Shirkey and Hammond, 2014; Snyder et al., 1982; Storz et al., 2010; Tate et al., 2020; Van Sant and Hammond, 2008; Velotta et al., 2016; Wearing et al., 2021, 2022; Wearing and Scott, 2022a; West et al., 2021). Our current findings add to growing evidence that high-altitude deer mice have also evolved changes in cardiovascular control by the sympathoadrenal system. The present study suggests that highland deer mice have evolved a more pronounced baroreflex, likely in association with greater cardiac responsiveness to β_1_-adrenergic receptor stimulation (Wearing et al., 2022), and they better maintain arterial blood pressure in hypoxia. The precise maintenance of blood pressure is essential for safeguarding tissue perfusion pressure and O_2_ delivery in hypoxia, and may thus contribute to fitness given the vital importance of thermogenic capacity to survival at high altitude (Hayes and O'Connor, 1999). However, highland deer mice also have lower circulating catecholamines and their adrenal medulla releases fewer catecholamines in response to stimulation (Pranckevicius et al., 2025; Scott et al., 2019).

Taken together, these findings suggest that high-altitude adaptation has led to a nuanced fine-tuning of the neural and humoral components of the sympathoadrenal system. Specifically, highland deer mice appear to have reduced the broad systemic effects of adrenal catecholamine release into the blood in chronic hypoxia, reducing potential issues related to prolonged sympathoexcitation (e.g. general systemic vasoconstriction and blood flow restriction to peripheral tissues) (Alvarez-Araos et al., 2024), while enhancing the autonomic neural control of blood pressure and cardiac output when needed to support high metabolic rates. These evolved changes in cardiovascular function may be especially important for coping with the metabolic challenges of living in the cold and hypoxic environment at high altitude.

## Supplementary Material

10.1242/jexbio.249483_sup1 Supplementary information
