# Supplementary material for: Evolved changes in reflex control of the cardiovascular system in deer mice native to high altitude
Source: J Exp Biol. 2025 Jun 18;228(12):jeb249483. doi: 10.1242/jeb.249483 (PMC12211558; doi:10.1242/jeb.249483)
Supplement: Supplementary information [file jexbio-228-249483-s1.pdf]

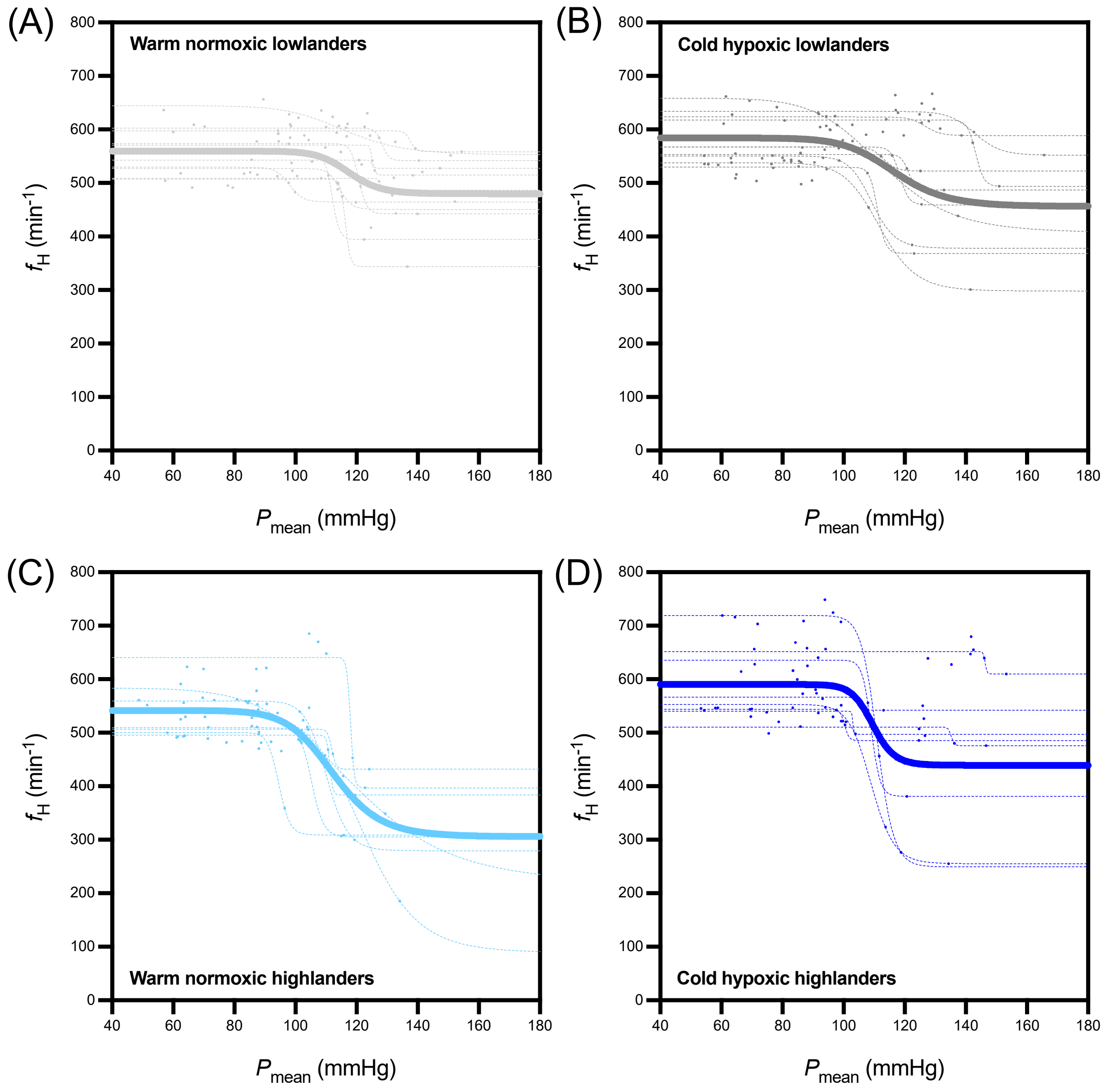

Fig. S1. Baroreflex curves for individual mice (thin lines and associated data points) and the average baroreflex curve for each group (bold solid curves). (**A**) warm normoxic lowlanders (n = 11 mice), (**B**) cold hypoxic lowlanders (n = 10 mice), (**C**) warm normoxic highlanders (n = 8 mice), and (**D**) cold hypoxic highlanders (n = 8 mice). See Materials and Methods for curve fitting methods and other details.

Journal of Experimental Biology • Supplementary information
